# Supplementary material for: Phase-dependent Andreev molecules and superconducting gap closing in coherently-coupled Josephson junctions
Source: Nat Commun. 2023 Dec 13;14:8271. doi: 10.1038/s41467-023-44111-3 (PMC10719386; doi:10.1038/s41467-023-44111-3)
Supplement: Supplementary file 1 — Supplementary Information [file 41467_2023_44111_MOESM1_ESM.pdf]

Supplementary information for "Phase-dependent Andreev molecules and superconducting gap closing in coherently coupled Josephson junctions"

Supplementary Note 1: Reproducibility of the AMS results

To confirm the reproducibility of our AMS results, we first warmed up the device and cooled it down again. This thermal cycle changes the impurity distribution in the semiconductors, resulting in the different properties of disorders. Then we performed the same measurement as we discussed in Figs. 2 in the main text. The results are shown in Fig. S1. Figures S1 (a) and (b) indicate the tunnel spectroscopic results of the single JJL and JJR using QPCL and QPCR, respectively. Figures S1(c) and (d) exhibit the tunnel spectroscopic results of the coupled JJL and JJR, respectively. As seen in these, the ABS periodic oscillation is obtained in the single JJ cases and the same modulation of the ABSs by the coherent coupling as seen in Figs. 2 on the main text appears in the coupled JJ cases. The results are noisier than those in the 1st cooldown. This is probably because the carrier mobility was degraded due to the thermal cycle and the QPCs became more unstable.

Secondly, we performed additional measurements to see the reproducibility using a different device with the same structure placed in our different dilution refrigerator. Then we obtained Fig. S2 as a result. The left and right figures exhibit the tunnel conductance G as a function of V and B in the single and coupled JJR. The left figure indicates the same periodic ABS oscillation as seen in Fig. 2(b). The right figure also shows the same remarkably modulated subgap structures from the ABSs in the single JJR as found in Fig. 2(d). Therefore, we confirm that our finding of the phase-dependent AMSs is reproducible.

Supplementary Note 2: Tunnel QPCs stability

As represented in the data jump found in Figs. 2, 4, and S1, our QPCs were not stable enough to maintain the proper condition for the tunnel spectroscopy due to the charge jumps and carrier accumulation over a long time. Therefore, we tuned the QPC conditions every time before starting the tunnel spectroscopic measurement. Typically, we tuned the gate voltages on the two QPCs around -5.5 V for the tunnel spectroscopy and around -6.5 V for pinching off the QPCs. For the tuning, we controlled the tunnel conductance of G_{L} and G_{R} around $\sim 0.1e^2/h$ and started sweeping the parameters such as bias voltage of V and magnetic field B.

Supplementary Note 3: Gate conditions for switching JJL and JJR

In the main text, we turned on and off the planar JJs of JJL and JJR. For the switching, we changed the local gate voltages V_{gL} and V_{gR} on the respective JJs. When we turned on the JJs, we set the gate voltages to 0 V. When we pinched off the JJs, we fixed the voltages at -6 V. As seen in Fig. S3(b) and (c), -6 V is sufficiently negative to remove the carriers in JJR. Furthermore, the local gate control result of JJL in Fig. S5(f) indicates that JJL is also pinched off around $V_{\mathrm{gL}} \sim -2$ V. Therefore, -6 V is also sufficiently negative to switch off JJL.

Supplementary Note 4: Local gate dependence of the JJR spectroscopic results

The local gate dependence of the coupled JJR spectroscopic results is shown in Figs. S3. For the measurement, we turned on JJL with $V_{\mathrm{gL}} = 0\ V$. Figure S3(a) is G_{R} as a function of V and B at V_{gR}= -0.5, -1.0, -1.5, and -2.0 V from the left to the right. The results maintain the AMS spectroscopic properties even at V_{gR}= -2.0 V. To confirm this, we checked G_{R} as a function of V and V_{gR} at B = -0.03 mT (a superconducting (SC) gap closing point) as shown in Fig. S3(b). No gap structure appears in the figure, which means that the AMSs are formed even when the local JJ is tuned. In addition, we measured G_{R} as a function of V_{gR} and B at V=0 mV as shown in Fig. S3(c). The two vertical lines corresponding to the SC gap closing points gradually disappear as V_{gR} is made more negative. This supports that the AMS is constructed even when the JJR is locally tuned. With the nonlocal gate control results in Figs. 4, we can conclude that the AMSs are formed by the coherent coupling of the asymmetric JJs.

Supplementary Note 5: Local and nonlocal gate dependence of the JJL spectroscopic results

To support the conclusion in supplementary note 6, we checked the consistency in the JJL spectroscopic results. For this sake, we implemented the tunnel spectroscopy of the coupled JJL at V_{gL}= 0 V with the QPCL. Figure S4 indicates the nonlocal gate control results. The represented results hold the similar properties observed in the nonlocal gate control results of the coupled JJR in Figs. 4. Figure S5 exhibits the local gate control results obtained with V_{gR}= 0 V. As well as Fig. S3(a), the AMS properties can be discovered in the negative V_{gL}. We note that Fig. S5(f) does not represent the SC gap closing because the magnetic field offset appears and the zero magnetic field value was slightly shifted while we were measuring these local gate control results. The consistency between the local and nonlocal gate control dependence of JJL and JJR supports that the coherent coupling occurs between the asymmetric JJs to form the AMSs. We note that Fig. S5(f) indicates that JJL is pinched off around $V_{\mathrm{gL}} \sim -2$ V. This behavior is consistent with the nonlocal gate control results shown in Figs. 4 in which the AMS properties disappear for $V_{\mathrm{gL}} < \sim -2$ V.

Supplementary Note 6: Robustness of the SC gap closing

To explore how robust the SC gap closing is, we indicate several line profiles obtained at the B points giving the locally maximal and minimal SC gap of Figs. 2(a) and (b), in blue and red in Figs. S6 (c) and (d), respectively. In Fig. 2(b), there is no charge jump in the data so we show the line profiles as they are. On the other hand, there are some jumps in Fig. 2(a), which change the tunnel conductance. To discuss the subgap structures, we normalized G_{L} by $G_{\mathrm{L}} = 0.200$ mV and the normalized G_{L} of the line profiles. As seen, the coupled JJR result in Fig. S6(d) indicates that the SC gap closing is found in all the red curves while all the blue curves are gapped. The coupled JJL result in Fig. S6(c) also indicates the gap closing behavior.

Supplementary Note 7: Model for numerical calculation

Here, we explain a model for numerical calculation based on a tight-binding method, which discretizes real space in a square lattice [1]. The ABSs are described by the Bogoliubov-de Gennes equation,

$$\begin{pmatrix} H - E_{\mathrm{F}} & \Delta(\boldsymbol{r}) \\ \Delta^{\dagger}(\boldsymbol{r}) & -(H^{*} - E_{\mathrm{F}}) \end{pmatrix} \begin{pmatrix} \psi_{\mathrm{e}} \\ \psi_{\mathrm{h}} \end{pmatrix} = E \begin{pmatrix} \psi_{\mathrm{e}} \\ \psi_{\mathrm{h}} \end{pmatrix}. \tag{S1}$$

Here, $H = H_0 + H_{\mathrm{SO}}$ is the Hamiltonian for electrons, where $H_0 = \boldsymbol{p}^2/(2m^*) + V(\boldsymbol{r})$ andH_{SO} the SOI Hamiltonian (see below) and E_{F} is the Fermi energy. $\Delta(\boldsymbol{r}) = \Delta_{\eta}(\boldsymbol{r}) \begin{pmatrix} 0 & -1 \\ 1 & 0 \end{pmatrix}$ describes the SC pair potentials in the left, shared, and right SC regions. ψ_{e} and ψ_{h} are spinors for an electron and a hole, respectively. On the two-dimensional tight-binding model, the differential operator in space is replaced into a hopping to neighboring lattice sites as

$$H_0 = t \sum_{j,l} (4 + v_{j,l}) c_{j,l}^{\dagger} c_{j,l} - \left(c_{j,l}^{\dagger} c_{j+1,l} + c_{j,l}^{\dagger} c_{j,l+1} + \mathrm{H.c.} \right) \tag{S2}$$

Here, $c_{j,l}^{\dagger}$ and $c_{j,}$ are the creation and annihilation operators of an electron at the site (j, l) indicating x and y directions, respectively. $v_{j,l}$ is an on-site potential describing the electron confinement and impurities. For the Rashba SOI, the Hamiltonian $H_{\mathrm{SO}} = (\alpha/\hbar)(-p_x \sigma_y + p_y \sigma_x)$ becomes [2]

$$\widehat{H}_{\mathrm{SO}} = i k_{\alpha} a t \sum_{j,l} \left(-\sigma_y c_{j,l}^{\dagger} c_{j+1,l} + \sigma_x c_{j,l}^{\dagger} c_{j,l+1} + \mathrm{H.c.} \right), \tag{S3}$$

where σ_x and σ_y are the Pauli matrices. $t = \hbar^2/(2m^* a^2)$ and a mean an energy unit and a lattice constant, respectively. The Rashba SOI strength is $k_{\alpha} = m^* \alpha a \hbar^2$.

The coupled JJs are modeled as shown in Fig. S7(a). Three yellow-shadowed regions are the SC regions, where the SC pair potentials, $\Delta_{\eta} = \Delta_0 e^{i\theta_{\eta}}$ (η = l,s,r), are given due to the SC proximity effect from the Aluminum to the two-dimensional electrons in the InAs quantum well in our experiments. In our consideration, the shared SC phase is fixed at $\theta_{\mathrm{s}} = 0$ and the two SC phase differences are defined as $\phi_{\mathrm{L}} = \theta_{\mathrm{s}} - \theta_{\mathrm{l}}$ and $\phi_{\mathrm{R}} = \theta_{\mathrm{r}} - \theta_{\mathrm{s}}$. Two white regions indicate the normal regions. We solve the Bogoliubov-de Gennes equation on this model and obtain the Andreev spectra. The parameters for the simulation are listed below. Several lengths denoted in Fig. S7(a) are $L_{\mathrm{w}} = (N_{\mathrm{w}} + 1)a, L_{\mathrm{s}} = (N_{\mathrm{s}} + 1)a, L_{\mathrm{nl}} = L_{\mathrm{nr}} = N_{\mathrm{n}} a$, $L_{\mathrm{l}} = N_{\mathrm{l}} a$, and $L_{\mathrm{r}} = N_{\mathrm{r}} a$. $N_{\mathrm{w}} = 29$, $N_{\mathrm{s}} = 7$, $N_{\mathrm{n}} = 4$, and $N_{\mathrm{l}} = N_{\mathrm{r}} = 20$ are the site numbers on the tight-binding model for the strongly coupled JJs. We change $N_{\mathrm{S}} = 49$ for the weakly coupled JJs to obtain Fig. 3(c). We use $L_{\mathrm{nl}} = 4a, N_{\mathrm{l}} = 20,$ and $N_{\mathrm{r}} = 20$ with $L_{\mathrm{s}} = 0$ and $L_{\mathrm{nr}} = 0$ for the single JJs. The lattice constant is $a = 20$ nm. The energy unit is $t \simeq 4.141$ meV for the effective mass in InAs $m^*= 0.023 m_{\mathrm{e}}$. The Fermi energy is set as $E_{\mathrm{F}} = 0.9t$ for the number of conduction channels $N_{\mathrm{ch}} = 9$ and $E_{\mathrm{F}} = 0.13t$ for $N_{\mathrm{ch}} = 3$. The SC pair potential is $\Delta_0 = 0.03t$. At the boundaries of SC regions, the strength of pair potential is divided by two. The strength α of Rashba SOI is given as $k_{\alpha} a = m^* \alpha a/\hbar^2 = 0.25$, which corresponds to $\alpha \simeq 4.14 \times 10^{-11}\ \mathrm{eV \cdot m}$, comparable to that in the InAs quantum wells. We give on-site random potentials $v_{j,l}$ to describe the effect of impurities at the sites $(x, y) = (ja, la)$. The range of randomness is $W_0/2 \geq v_{j,l} \geq -W_0/2$ with $W_0 = 2t,$ which corresponds to the mean free path $l_{\mathrm{mfp}} \simeq 217$ nm.

The typical Andreev spectra of the strongly coupled JJs are shown in Figs. 3(e), (g), (h), and (i) in the main text, where the Fermi energy is $E_{\mathrm{F}} = 0.9t$ with the on-site random potential of $W_0 = 2t$.

To evaluate the probability that the zero energy states emerge along $\phi_{\mathrm{L}} = \phi_{\mathrm{R}}$, we examine 200 or 201 samples with different impurity strength and Rashba SOI strength at $E_{\mathrm{F}} = 0.9t$. The results are summarized in Fig. S7(b). The probability is 0% with no SOI strength and increases up to about 20% as the SOI strength increases. This probability is high enough to see the zero energy states in experiments in the presence of impurity scattering. In the present range of impurity strength, the influence of impurity is almost negligible for the emergence of zero energy states.

Figures S7(c-f) exhibit the Andreev spectra at $E_{\mathrm{F}} = 0.9t$ (c,d) and $0.13t$ (e,f) with no impurity ($W_0 = 0$). We put an index for the ABSs by $n = \pm 1, \pm 2, \pm 3, \cdots$. The lowest positive and highest negative ABSs are $n = 1$ and -1, respectively. In Figs. S7(d) and (f), the same colors represent the same $|n|$.

Supplementary Note 8: Fermi energy tuning

Here, we study the Andreev spectra of the strongly coupled JJs with the different Fermi energies. Figures S8(a) and (b) represent dispersion relations of conduction electrons on the tight-binding model with only $-(\alpha/\hbar)p_x\sigma_y$ of the dominant term in the Rashba SOI and with $(\alpha/\hbar)(-p_x\sigma_y + p_y\sigma_x)$ of the full Rashba SOI, respectively. Note that the dispersions are shifted to positive by $E_{\mathrm{SO}} = \hbar^2 k_\alpha^2/(2m^*) = t(k_\alpha a)^2$ to avoid negative energy. If one considers only the dominant term in the Rashba SOI, the dispersions are spin-split in the k_x direction and the up and down spins in the y direction are well defined (Fig. S8(a)). Subbands formed by a confinement in the y direction are not hybridized. In this case, the Fermi velocities do not depend on the spin directions. When the Rashba SOI is fully considered in Fig. S8(b), the $p_y\sigma_x$ term mixes the subbands, resulting in the spin-dependent Fermi velocities [3,4]. The spin-dependent Fermi velocities can produce the spin-split Andreev spectra in the JJs [5] and the zero energy states following the scenario discussed in Fig. 3 in the main text. A previous theoretical study for a similar structure [6] also pointed out that the difference of the Fermi velocities for the up and down spins is essential to obtain the zero energy states consistent with topological transition.

For $E_{\mathrm{F}} = 0.9t$ and $0.13t$, we study the spectra with $E_{\mathrm{F}} + \delta$ in a range $-0.03t \leq \delta \leq 0.03t$ and the obtained positive lowest ABS energy E_1 is shown in Figs. S8(c) and (d), respectively. We set $W_0 = 0$ for the calculation. The considered range of Fermi energy is indicated with blue lines in Fig. S8(b). At $E_{\mathrm{F}} \approx 0.9t$, a difference of the Fermi velocities for the up and down spins is not large. On the other hand, at $E_{\mathrm{F}} \approx 0.13t$, the difference is significant. The Andreev spectra at $E_{\mathrm{F}} \approx 0.13t$ in Fig. S8(d) exhibit the clearer emergence of zero energy states than those in Fig. S8(c) at $E_{\mathrm{F}} \approx 0.9t$. This implies that the zero energy states would be more detectable in the lower carrier density because the modulation of the Fermi velocities is larger.

Supplementary Note 9: Spin polarization of AMSs

To confirm the zero energy states, we examine the spin polarization of the AMSs in the strongly

coupled JJs around the zero energy crossing. The spin-split AMSs are formed by the electrons with up (down) spin and holes with down (up) spin. Therefore, we introduce spin operators for the AMSs as

$$S_\xi = \begin{pmatrix} \sigma_\xi & 0 \\ 0 & -\sigma_\xi^* \end{pmatrix} \quad (\xi = x, y, z) \tag{S4}$$

with the Pauri matrices $\sigma_x, \sigma_y, \sigma_z$ for electrons. For the wavefunction of the AMSs $\Psi_n = (\psi_\mathrm{e}, \psi_\mathrm{h})^\mathrm{t} = (\psi_{\mathrm{e}\uparrow}, \psi_{\mathrm{e}\downarrow}, \psi_{\mathrm{h}\uparrow}, \psi_{\mathrm{h}\downarrow})^\mathrm{t}$, the expectation values of the spin polarization are evaluated as

$$\langle S_{n,x} \rangle = \Psi_n^\dagger S_x \Psi_n = 2|\psi_{\mathrm{e}\uparrow}\psi_{\mathrm{e}\downarrow}| \cos(\varphi_{\mathrm{e}\downarrow} - \varphi_{\mathrm{e}\uparrow}) - 2|\psi_{\mathrm{h}\uparrow}\psi_{\mathrm{h}\downarrow}| \cos(\varphi_{\mathrm{h}\downarrow} - \varphi_{\mathrm{h}\uparrow}), \tag{S5}$$

$$\langle S_{n,y} \rangle = \Psi_n^\dagger S_y \Psi_n = 2|\psi_{\mathrm{e}\uparrow}\psi_{\mathrm{e}\downarrow}| \sin(\varphi_{\mathrm{e}\downarrow} - \varphi_{\mathrm{e}\uparrow}) + 2|\psi_{\mathrm{h}\uparrow}\psi_{\mathrm{h}\downarrow}| \sin(\varphi_{\mathrm{h}\downarrow} - \varphi_{\mathrm{h}\uparrow}), \tag{S6}$$

$$\langle S_{n,z} \rangle = \Psi_n^\dagger S_z \Psi_n = |\psi_{\mathrm{e}\uparrow}|^2 - |\psi_{\mathrm{e}\downarrow}|^2 - |\psi_{\mathrm{h}\uparrow}|^2 + |\psi_{\mathrm{h}\downarrow}|^2. \tag{S7}$$

Here, φ_ζ is the phase of ψ_ζ.

Figure S9(a) shows a log-scale plot of the positive AMSs at $E_\mathrm{F} = 0.9t$. Here, we do not consider the impurity ($W_0 = 0$). The positive lowest AMS ($n = 1$) indicates extensively sharp dips at $\phi_\mathrm{L} = \phi_\mathrm{R} \cong 0.5784\pi$ and $\cong 0.5883\pi$. The negative highest level ($n = -1$) also indicates sharp peaks at the same position.

Figure S9(b) exhibits the spin polarization of $n = \pm 1$. We find no spin polarization in the x- and z-directions, $\langle S_{\pm 1,x} \rangle = 0$ and $\langle S_{\pm 1,z} \rangle = 0$, whereas the spin polarization in the y-direction is finite. It is attributed to a dominant contribution of $-(\alpha/\hbar)p_x\sigma_y$ in the Rashba SOI. Since the positive and negative levels satisfy the electron-hole symmetry, we find $\langle S_{n,y} \rangle = -\langle S_{-n,y} \rangle$. At the zero energy crossing points, the spin polarizations of $n = \pm 1$ AMSs are flipped. This indicates the level crossing, namely the presence of zero energy states. Figures S9(c) and (d) exhibit the zero energy crossing behaviors and the spin polarization at $E_\mathrm{F} = 0.13t$. The spin polarization is larger than that of $E_\mathrm{F} = 0.9t$. It might be attributed to a larger factor $E_\mathrm{SO}/E_\mathrm{F}$.

Supplementary Note 10: Allowed region for zero energy states

In the main, we discuss the SC gap closing in the tunnel spectroscopy measurement when the AMS is formed in the coupled JJs. In the experiment, the spectroscopy is performed with $\phi_\mathrm{L} = \phi_\mathrm{R}$ and the gap closing behavior is found at $\pm\phi_0$ shifted from $\phi_\mathrm{L} = \phi_\mathrm{R} = \pi$ (see Figs. 2(c) and (d)). A numerical calculation with the strong SOI supports the emergence of zero energy states.

In Ref. [6], Lesser et al. discussed two cases of SNSNS junctions with no impurity. Here, the SC phases are set as $\theta_\mathrm{l} = -\phi_\mathrm{L}$, $\theta_\mathrm{s} = 0$, and $\theta_\mathrm{r} = \phi_\mathrm{R}$ for the left, center, and right superconductors. First, they have considered a decrease of SC pair potential of the center SC region. Then, the junction becomes like a single JJ and the zero energy states are allowed only when $\phi_\mathrm{L} + \phi_\mathrm{R} = (2n + 1)\pi$ with an integer n [8]. Second, they consider when the center SC region is very wide and the ABSs in the two normal regions are separated enough. Then, the system is regarded as the separated two JJs, and the zero energy states are allowed on $\phi_\mathrm{L} = (2n + 1)\pi$ or $\phi_\mathrm{R} = (2n + 1)\pi$. If the very wide SNS junction for the first case is gradually modulated to the very wide SNSNS junction for the second case, the condition $\phi_\mathrm{L} + \phi_\mathrm{R} = \pi$ may be continuously changed to $\phi_\mathrm{L} = \pi$ and $\phi_\mathrm{R} = \pi$ in the range of $-\pi/2 \leq \phi_\mathrm{L}, \phi_\mathrm{R} \leq 3\pi/2$. Therefore, for general SNSNS junctions, the zero energy states can be

allowed only inside the shaded triangles by the three lines in Fig. S10(a).

In Ref. [7], van Heck et al. have theoretically considered the Andreev spectra in multiterminal JJs. Based on the scattering matrix approach [9], they have discussed a lower boundary of the spectra and the existence of zero energy states in the short junction limit. A JJ with several superconductors is described in terms of the scattering matrix as

$$s_{\mathrm{A}} s_{\mathrm{N}} \Psi = \Psi \tag{S8}$$

with the scattering matrix for the normal region

$$s_{\mathrm{N}} = \begin{pmatrix} s_0 & 0 \\ 0 & s_0^* \end{pmatrix} \tag{S9}$$

and for the Andreev reflection

$$s_{\mathrm{A}} = \alpha_{\mathrm{A}}(\epsilon) \begin{pmatrix} 0 & r_{\mathrm{A}}^* \\ r_{\mathrm{A}} & 0 \end{pmatrix}. \tag{S10}$$

Here, Ψ is an eigenmode for the ABSs. s_0 describes the electron transport and scattering in the normal region. Note that the scattering matrix satisfies $s_0^\dagger s_0 = 1$. $\alpha_{\mathrm{A}}(\epsilon) = \sqrt{1-\epsilon^2} + i\epsilon$ is a phase factor with $\epsilon = E/\Delta_0$. r_{A} is a diagonal matrix describing the SC phases, $r_{\mathrm{A}} = \mathrm{diag}\left(ie^{i\theta_1}, ie^{i\theta_2}, \cdots, ie^{i\theta_m}\right)$ with the number of SC regions m. After a short algebra, they obtain

$$\begin{pmatrix} 0 & -iA^\dagger \\ iA & 0 \end{pmatrix} \Psi = \epsilon \Psi \tag{S11}$$

with $A = (r_{\mathrm{A}} s_0 - s_0^{\mathrm{t}} r_{\mathrm{A}})/2$. Since A is a normal matrix ($AA^\dagger = A^\dagger A$), the eigenvalue problem is simplified as $A\psi = |\epsilon| e^{i\chi} \psi$ with a phase χ satisfying $\Psi = \left(\psi, e^{i\chi}\psi\right)^{\mathrm{t}}$. If the time-reversal symmetry is present for the normal region, the scattering matrix should be $s_0 = -s_0^{\mathrm{t}}$. The problem is rewritten as

$$s_0 r_{\mathrm{A}} \psi + r_{\mathrm{A}} \psi' = 2|\epsilon| e^{i\chi} \psi \tag{S12}$$

with $\psi' = s_0 \psi$. As a property of the scattering matrix, a norm of the vector is kept, $\|\psi\| = \|\psi'\| = 1$. By applying $\psi'^\dagger$ from the left and considering the Cauchy-Schwarz inequality, they find a lower boundary condition of

$$|\epsilon| \geq \frac{1}{2} \left| \psi^\dagger r_{\mathrm{A}} \psi + \psi'^\dagger r_{\mathrm{A}} \psi' \right|. \tag{S13}$$

Because r_{A} is the diagonal matrix of the SC phases $\{\theta_j\}$, they consider geometrical illustrations for $\left(\psi^\dagger r_{\mathrm{A}} \psi + \psi'^\dagger r_{\mathrm{A}} \psi'\right)/2$ as Figs. S10(b), where a polygon expanded by the phases $\{\theta_j\}$ provides the lower boundary $\epsilon_{\min}$. There are two possibilities: One is that the polygon does not cover the origin (left) and another case covers the origin (right). The former case has a lower boundary of the spectrum as $\epsilon_{\min} = \min_{i,j} \left[\cos\left(\left(\theta_i - \theta_j\right)/2\right)\right]$. The latter case allows the presence of zero energy states. This condition is redefined as a winding of the SC phases.

When we order as $\theta_1 \leq \theta_2 \leq \cdots \leq \theta_m$ and introduce $\phi_i = \theta_{i+1} - \theta_i \in (-\pi, \pi]$, the winding of the SC phases

$$N_{\text{win}} = \frac{1}{2\pi}\sum_i \phi_i \tag{S14}$$

becomes $N_{\text{win}} = 1$ for $\epsilon_{\text{min}} = 0$ and $N_{\text{win}} = 0$ for $\epsilon_{\text{min}} \neq 0$.

We fix $\theta_2 = 0$ and set $\theta_1 = -\phi_{\text{L}} \leq 0$ and $\theta_3 = \phi_{\text{R}} \geq 0$ for three-terminal JJ ($m = 3$) to consider our coupled planar JJs. Here, $\pi \geq \phi_{\text{L}}, \phi_{\text{R}} \geq 0$. The condition for $\epsilon_{\text{min}} = 0$ corresponds to $\phi_{\text{L}} + \phi_{\text{R}} \geq \pi$.

Supplementary Note 11: Absence of spin-orbit interaction

As a reference, we examine the Andreev spectra in a single JJ, weakly coupled JJs, and strongly coupled JJs in the absence of SOI in Fig. S11. The other parameters are the same as those used to obtain Figs. 3(a), (c), and (e) in the main text. The ABSs are spin-degenerated. In the isolated single JJ, the SC gap becomes minimal at $\phi_{\text{L}} = \pi$ or $\phi_{\text{R}} = \pi$ in Fig. S11(a). The weakly coupled AMSs hold the minimal SC gap at $\phi_{\text{L}} = \phi_{\text{R}} = \pi$ in Fig. S11(b). Unlike in the presence of the SOI, the spin-degenerated AMSs are hybridized and open the SC gap. Consequently, the spectra hold a maximum of the SC gap at $\phi_{\text{L}} = \phi_{\text{R}} = \pi$ and minima at $\phi_{\text{L}} = \phi_{\text{R}} = \pi \pm \phi_0$ with $\phi_0 \neq 0$.

Supplementary Figures

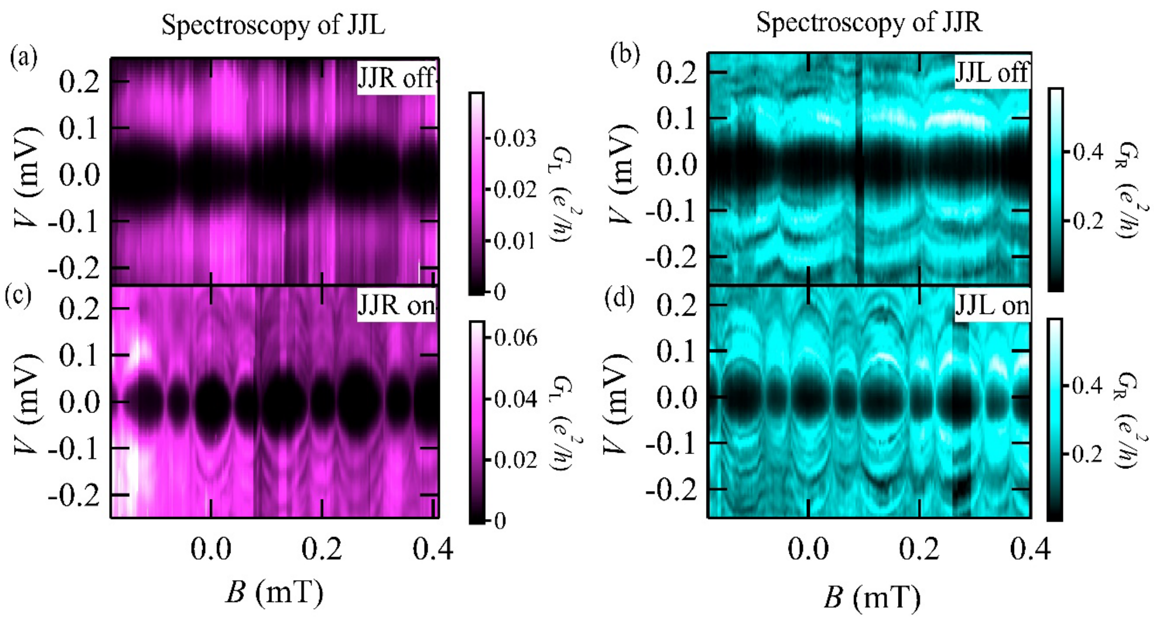

Fig. S1

The tunnel spectroscopic results obtained in the 2nd cooldown of the same device as we discussed in the main text. (a) and (b) exhibit the single JJL and JJR results, respectively. (c) and (d) exhibit the coupled JJL and JJR results, respectively. The SC gap oscillations assigned to the phase-dependent ABSs in the single JJs appear in (a) and (b). Additionally, the AMS features can also be found in (c) and (d).

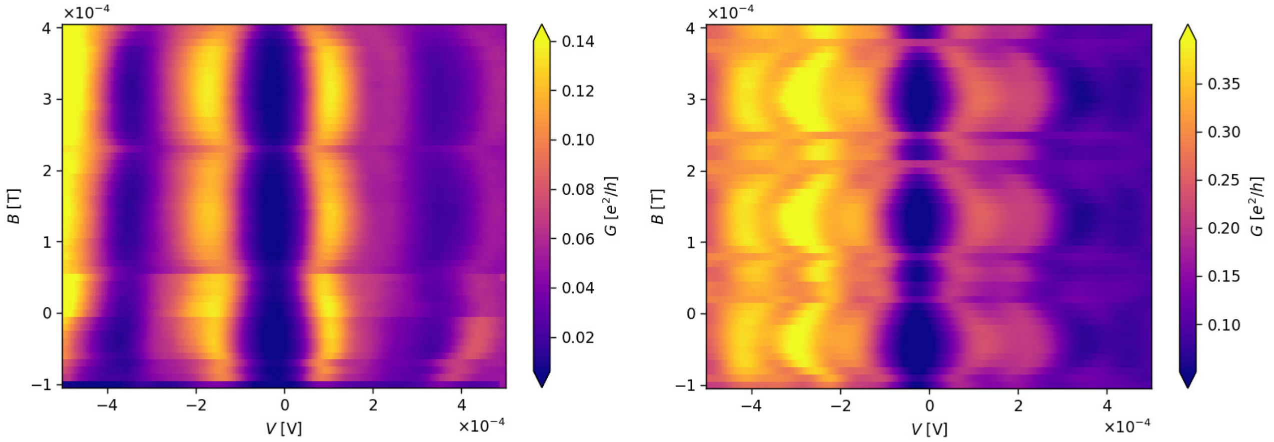

Fig. S2

The left indicates the differential conductance of the single JJR of the different devices measured in the different dilution fridge at 50 mK of the base temperature. The SC gap oscillation can be found. The right indicates the differential conductance of the coupled JJR. The AMS features appear.

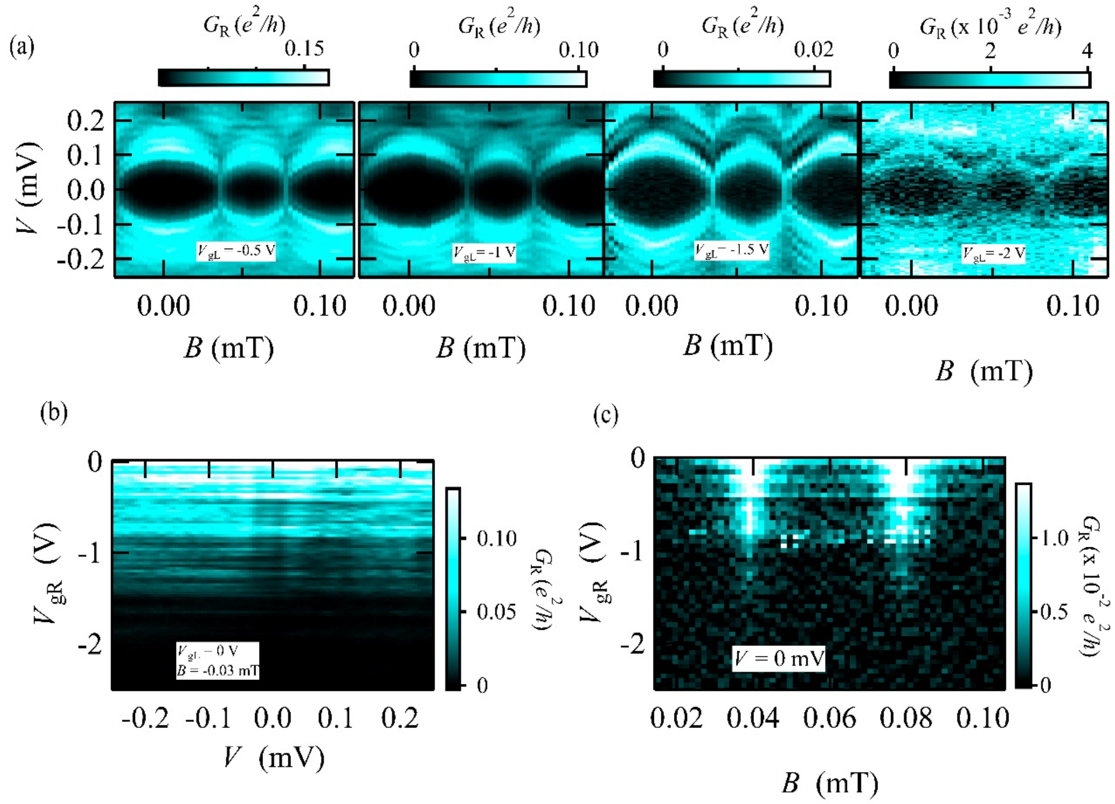

Fig. S3

(a) G_R as a function of V and B at different V_{gR}. Even when V_{gR} becomes more negative, the AMS features do not disappear. The results become vague because the negative V_{gL} also affects the tunnel barrier height of QPCR. (b) G_R as a function of V and V_{gR} at $V_{gL} = 0$ V and B = -0.03 mT which gives the minimum SC gap. (c) G_R as a function of V_{gR} and B at $V = 0$ mV. The SC gap closing lines appear bright around B ~0.04 and 0.08 mT.

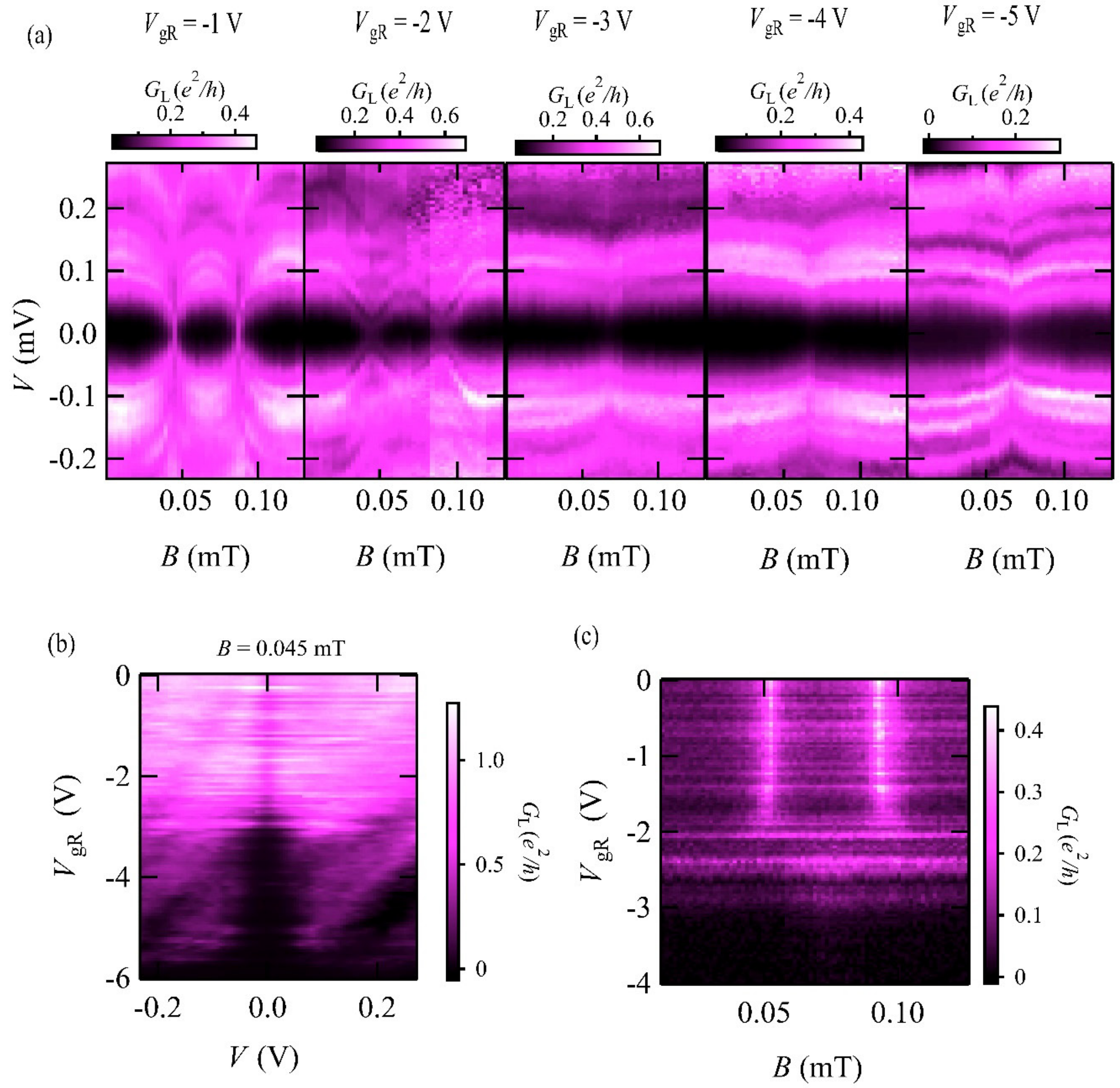

Fig. S4

(a) G_{L} as a function of V and B at different V_{gR}. When V_{gR} becomes more negative than -2 V, the AMS features disappear and the G_{L} characteristics reflect the single JJR properties as well as Fig. 4(a). (b) G_{L} as a function of V and V_{gR} at $V_{\mathrm{gL}} = 0$ V and B = 0.045 mT which gives to the minimum SC gap. (c) G_{L} as a function of V_{gR} and B at $V = 0$ mV. The SC gap closing lines appear bright around B ~0.05 and 0.08 mT.

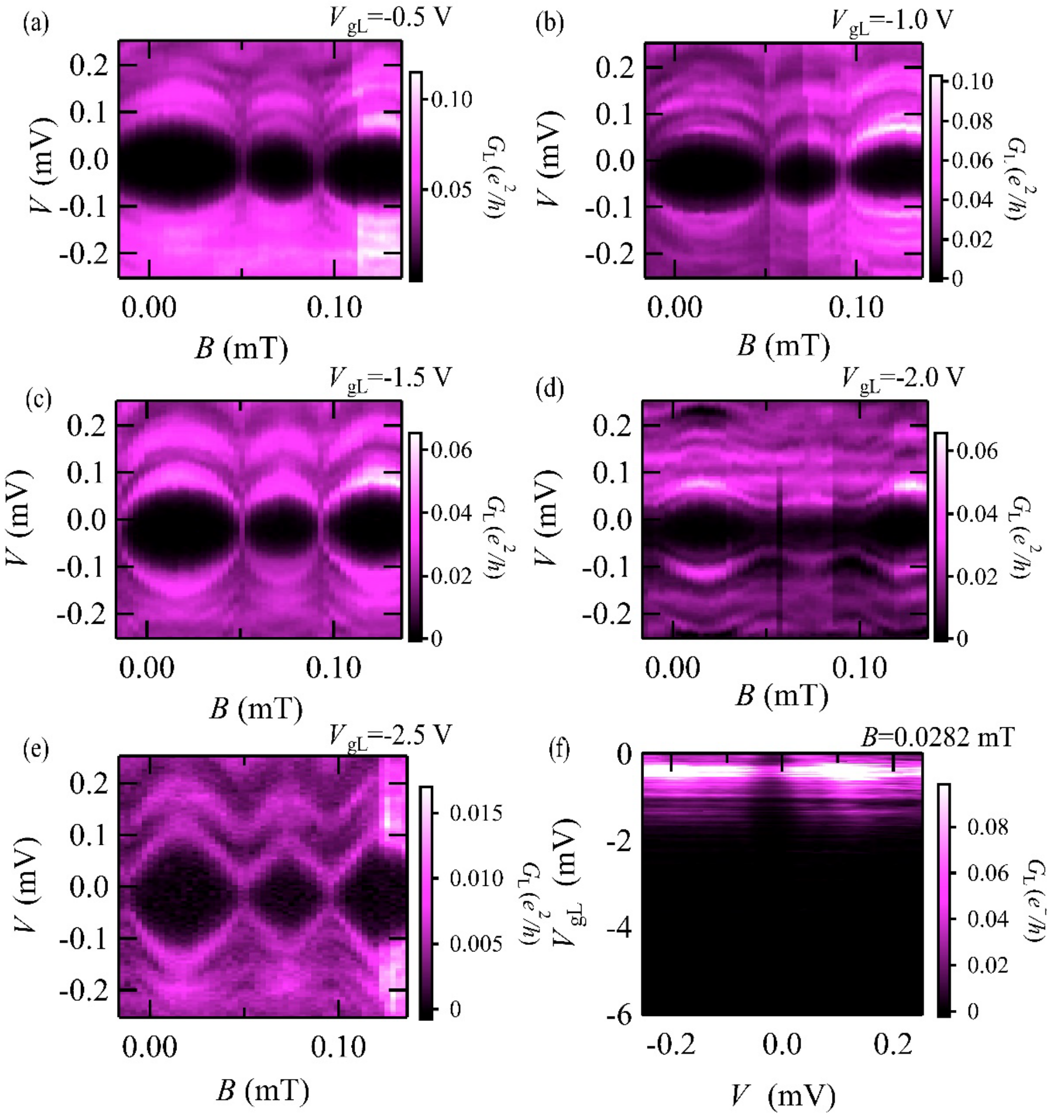

Fig. S5

(a), (b), (c), (d), and (e) exhibit G_L as a function of V and B at different V_{gL}. Even when V_{gL} becomes more negative, the AMS features do not disappear. The results become vague because the negative V_{gL} also affects the tunnel barrier height of QPCR. (b) G_L as a function of V and V_{gL} at $V_{gR} = 0$ V and B = 0.0282 mT which gives to the minimum SC gap.

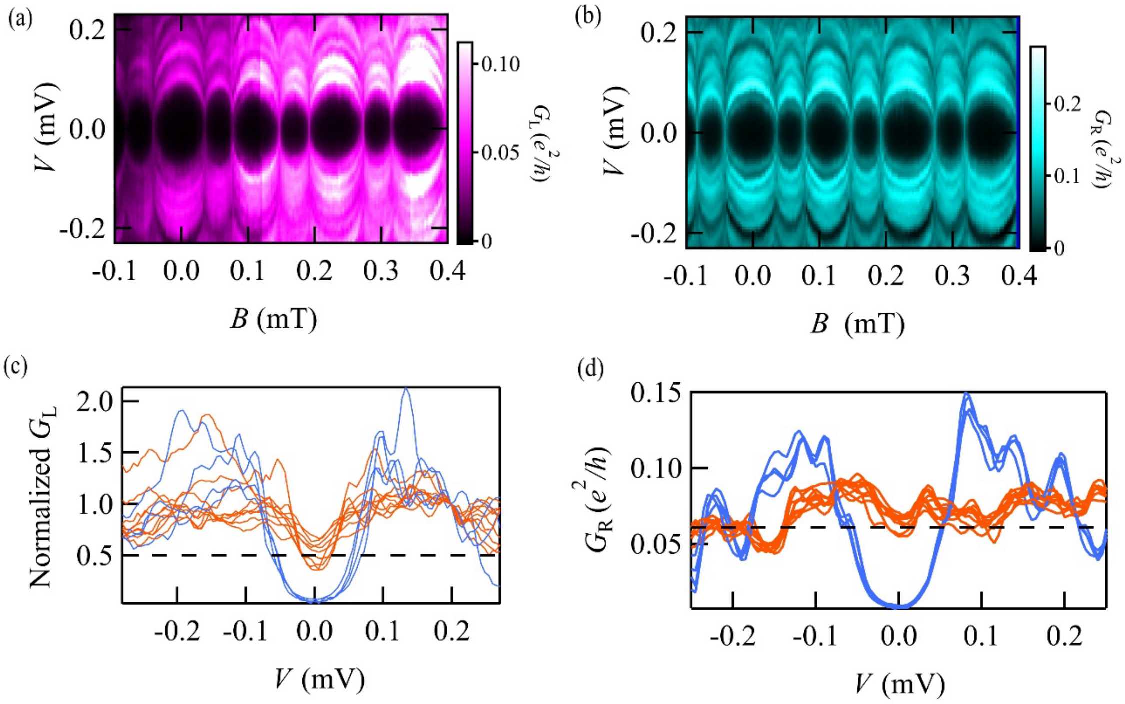

Fig. S6

(a) and (b) indicate the same results shown in Figs. 2(c) and (d). The blue and red lines in (c) show the line profiles at the B points giving the locally maximal and minimal SC gap of (a), respectively. To compare the blue and red lines, we normalize the line profiles by G_L at V=0.200 mV because the charge jumps change the QPCL condition in the measurement of (a). The blue and red lines in (d) show the line profiles at the B points giving the locally maximal and minimal SC gap of (b), respectively.

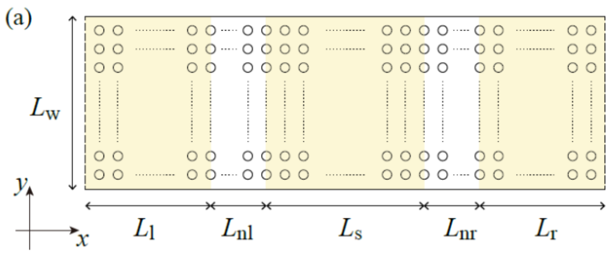

(b) Rashba SOI strength: $k_\alpha a$

impurity strength: W_0/t

	0.0	0.05	0.10	0.15	0.20	0.25	0.30
0.5	0.0%	5.0%	13.0%	12.0%	11.0%	19.9%	19.0%
1.0	0.0%	9.5%	12.0%	12.0%	14.0%	17.4%	19.0%
1.5	0.0%	6.0%	15.0%	14.0%	14.5%	17.9%	23.5%
2.0	0.0%	5.5%	13.9%	17.4%	19.9%	19.4%	20.5%

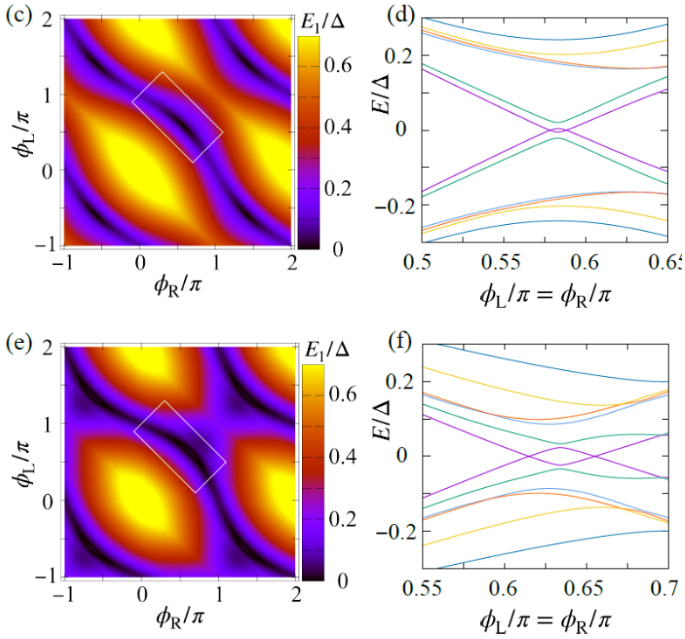

Fig. S7

Tight-binding model for coupled JJs described in a two-dimensional space. L_w, L_l, L_{nl}, L_s, L_{nr}, and L_r are the width of the system, the length of the left SC, left normal, shared SC, right normal, and right SC regions, respectively, with the lattice constant a. (b) Summary of emergence probability of the zero energy states when the SOI strength and impurity strength are tuned. (c,d) Andreev spectrum at a clean limit ($W_0 = 0$) when $E_F = 0.9t$ and (e,f) when $E_F = 0.13t$. White boxes in (c) and (e) indicate a plot range in Figs. S8(c) and (d).

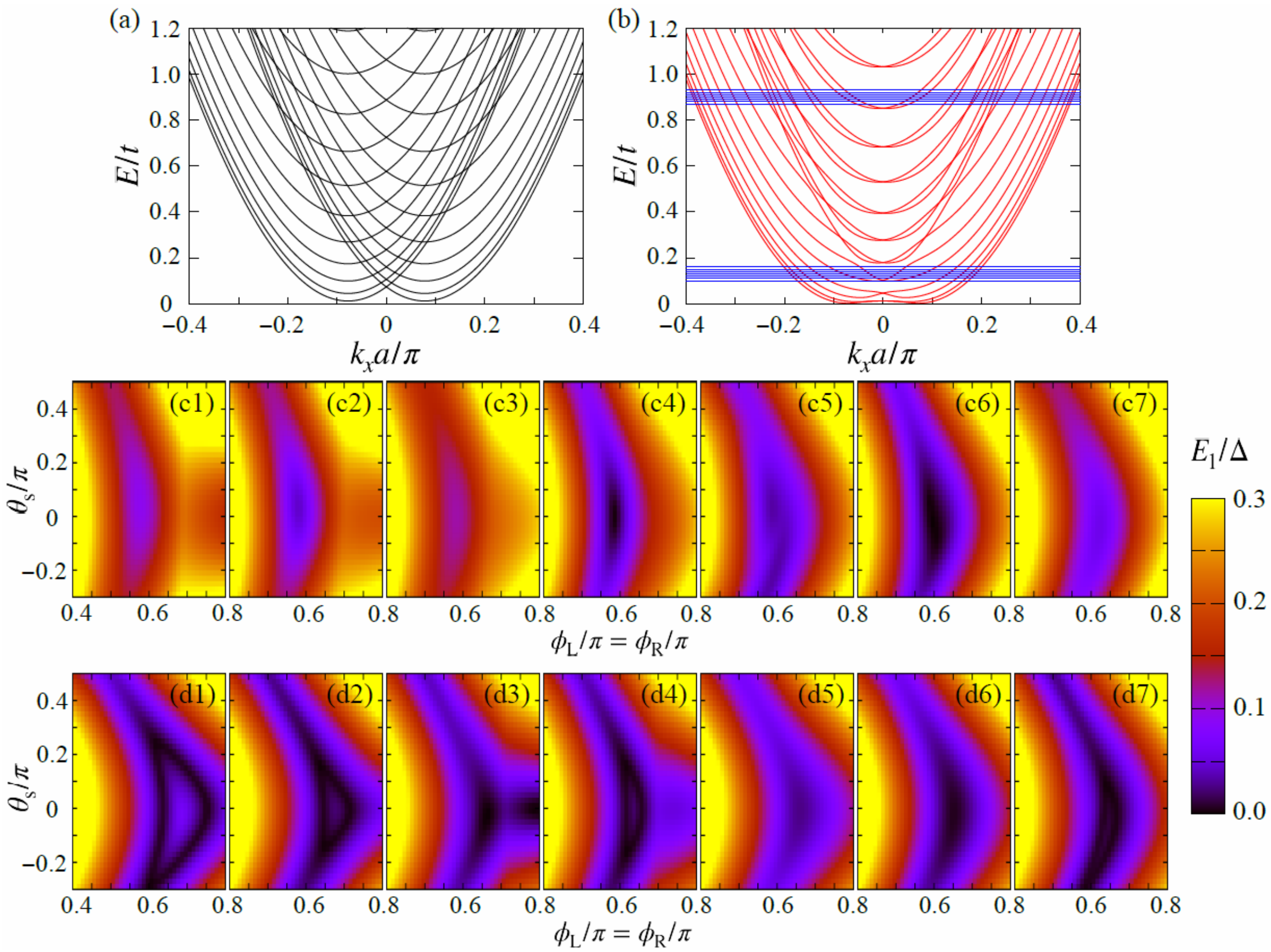

Fig. S8

(a,b) Dispersion relation $E(k_x)$ of electrons in the normal regions on the tight-binding model without $p_y\sigma_x$ term in Rashba SOI (a) and with full Rashba SOI (b). Blue thick lines in (b) indicate $E/t = 0.13$ and 0.90. Blue thin lines are $E/t = 0.13 + 0.01 \times n$ and $0.90 + 0.01 \times n$ ($n = \pm 1, \pm 2, \pm 3$). (c,d) Andreev spectra in white boxes in Fig. S7(c,e) when the Fermi energy is tuned slightly, $E_{\mathrm{F}} + \delta$, around $E_{\mathrm{F}}/t = 0.90$ (c) and 0.13 (d). The tuning range is $\delta/t = -0.03 \sim +0.03$. (c1)-(c7) and (d1)-(d7) are the spectra from the negative to positive tuning with 0.01t step. (c4) and (d4) are cases of $\delta = 0$.

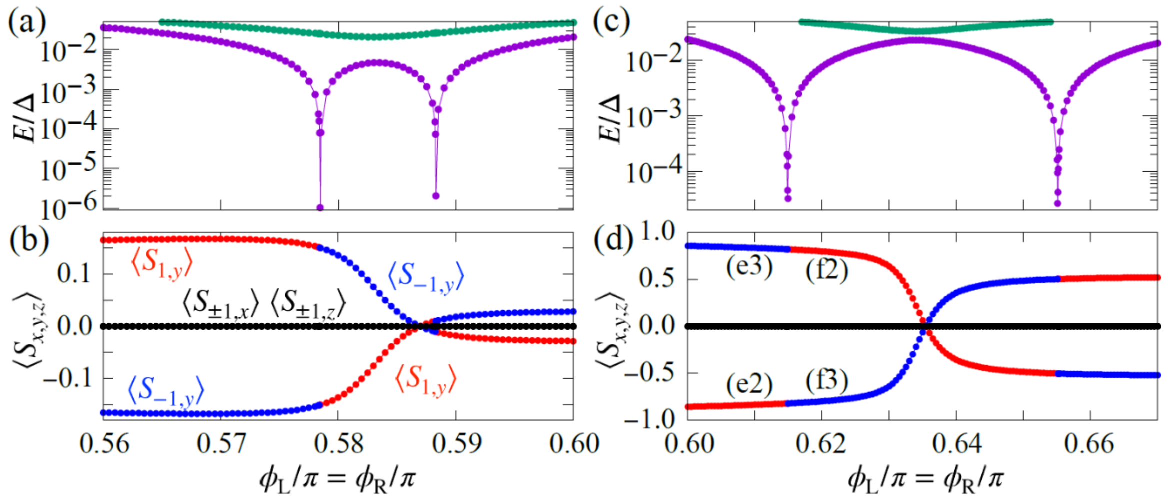

Fig. S9

(a) Logscale replot of the positive Andreev levels in Fig. S7(d) when $E_{\mathrm{F}} = 0.9t$. (b) Spin polarization $\langle S_{\pm,\xi}\rangle$ ($\xi = x, y, z$) of the positive lowest and negative highest Andreev levels in (a). (c) Logscale replot of the positive Andreev levels in Fig. S7(f) when $E_{\mathrm{F}} = 0.13t$. (d) Spin polarization $\langle S_{\pm,\xi}\rangle$ ($\xi = x, y, z$) of the positive lowest and negative highest Andreev levels in (c).

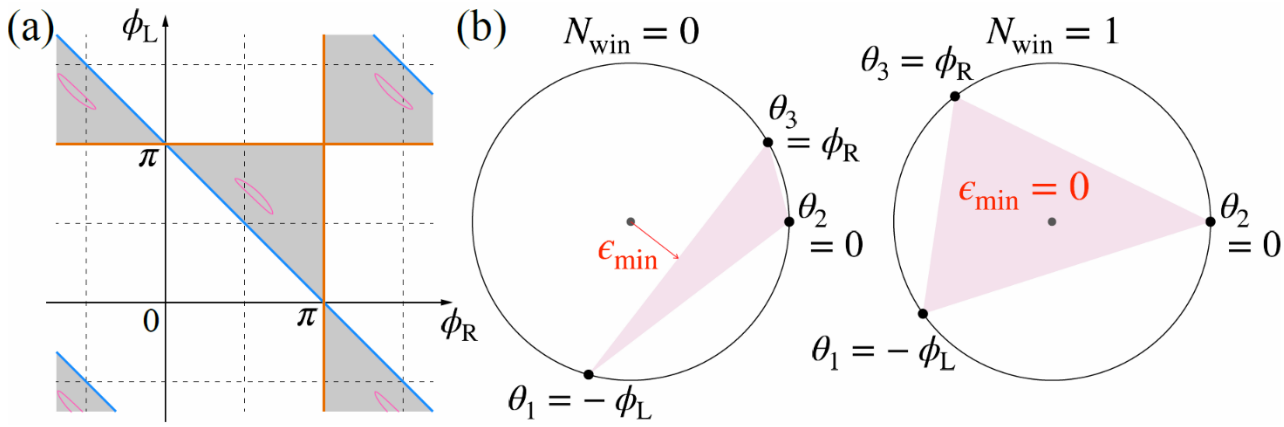

Fig. S10

(a) Allowed regions for zero energy states following the discussions in Refs. [6,7]. Pink curves indicate the zero energy crossing positions in our results in Fig. 3(i) where the Fermi energy is $E_{\mathrm{F}} = 0.9t = 3.727$ meV, the Rashba SOI is $k_{\alpha}a = 0.25$ corresponds to $\alpha \simeq 4.14 \times 10^{-11}\mathrm{eV}\cdot\mathrm{m}$ and the range of on-site random potentials is $W_0 = 2t$ corresponds to the mean free path being $l_{\mathrm{mfp}} \simeq 217\mathrm{nm}$. (b) Geometrical illustrations for $(\psi^{\dagger} r_{\mathrm{A}} \psi + \psi'^{\dagger} r_{\mathrm{A}} \psi')/2$ in case of three terminal JJ with $\theta_2 = 0$.

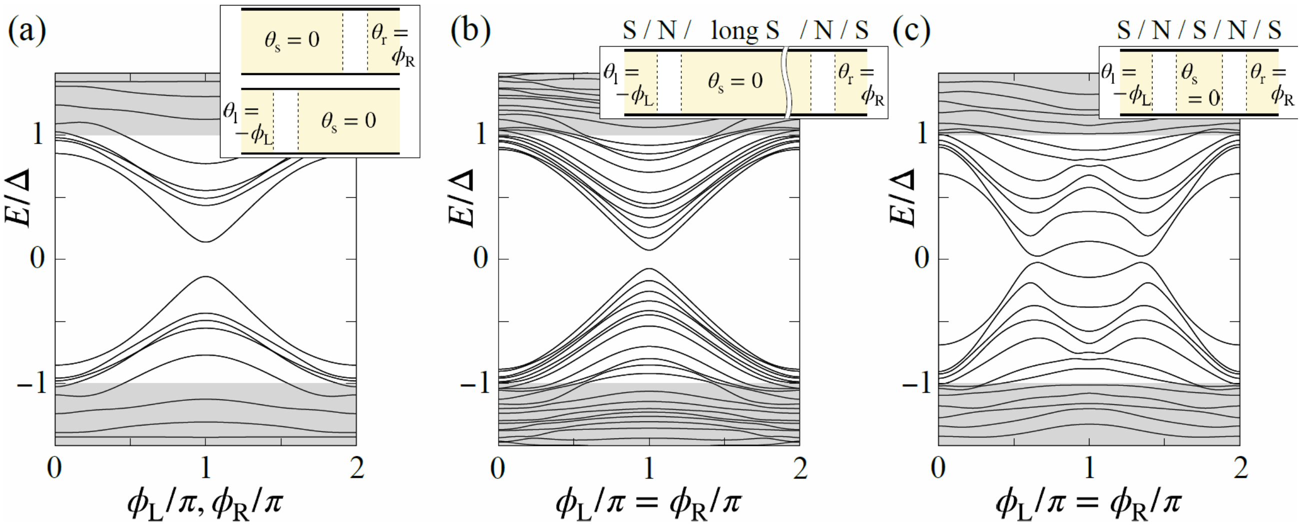

Fig. S11

Andreev spectra in the absence of SOI for single JJs in (a), a weakly coupled JJ in (b), and a strongly coupled JJ in (c). The SOI is $k_\alpha = 0$. The other parameters of the junctions are the same as those in Figs. 3(a), (c), and (e). The inset is a schematic image of the considered single JJ (isolated JJL or JJR). The yellow and white regions represent the SC electrode and the normal region, respectively. The phases of the SC electrodes are written.

Supplementary References

[1] S. Datta, *Electronic Transport in Mesoscopic Systems* (Cambridge University Press, Cambridge, 1995).

[2] T. Yokoyama, M. Eto, and Y. V. Nazarov, *Anomalous Josephson Effect Induced by Spin-Orbit Interaction and Zeeman Effect in Semiconductor Nanowires*, Physical Review B **89**, 195407 (2014).

[3] M. Governale and U. Zülicke, *Spin Accumulation in Quantum Wires with Strong Rashba Spin-Orbit Coupling*, Phys. Rev. B **66**, 073311 (2002).

[4] C.-H. Hsu, P. Stano, Y. Sato, S. Matsuo, S. Tarucha, and D. Loss, *Charge Transport of a Spin-Orbit-Coupled Luttinger Liquid*, Phys. Rev. B **100**, 195423 (2019).

[5] L. Tosi, C. Metzger, M. F. Goffman, C. Urbina, H. Pothier, S. Park, A. L. Yeyati, J. Nygård, and P. Krogstrup, *Spin-Orbit Splitting of Andreev States Revealed by Microwave Spectroscopy*, Phys. Rev. X **9**, 011010 (2019).

[6] O. Lesser, Y. Oreg, and A. Stern, *One-Dimensional Topological Superconductivity Based Entirely on Phase Control*, Phys. Rev. B **106**, L241405 (2022).

[7] B. van Heck, S. Mi, and A. R. Akhmerov, *Single Fermion Manipulation via SC Phase Differences in Multiterminal Josephson Junctions*, Physical Review B **90**, (2014).

[8] Y. V. Nazarov, Y. M. Blanter, *Quantum Transport: Introduction to Nanoscience* (Cambridge University Press, Cambridge, 2009).

[9] C. W. J. Beenakker, *Universal Limit of Critical-Current Fluctuations in Mesoscopic Josephson Junctions*, Phys. Rev. Lett. **67**, 3836 (1991).
